# Unusual Plastoquinones in Non‐Phototrophic Nitrifying Bacteria

**DOI:** 10.1111/1758-2229.70174

**Published:** 2025-08-05

**Authors:** Nicole J. Bale, Hayato Fujimura, Petra Pjevac, Michel Koenen, Hikaru Ikeda, Satohiro Itagaki, Yojiro Yamamoto, Johanna Palmetzhofer, Christopher J. Sedlacek, Hayk Palabikyan, Jaap S. Sinninghe Damsté, Michael Wagner, Hiroshi Shiigi, Holger Daims

**Affiliations:** ^1^ Department of Marine Microbiology and Biogeochemistry NIOZ Royal Netherlands Institute for Sea Research Den Burg the Netherlands; ^2^ Department of Applied Chemistry Osaka Metropolitan University Sakai Japan; ^3^ Centre for Microbiology and Environmental Systems Science University of Vienna Vienna Austria; ^4^ Doctoral School in Microbiology and Environmental Science University of Vienna Vienna Austria; ^5^ Biology Department University of Southern Indiana Evansville Indiana USA; ^6^ Department of Earth Sciences Utrecht University, Faculty of Geosciences Utrecht the Netherlands; ^7^ The Comammox Research Platform University of Vienna Vienna Austria; ^8^ Center for Microbial Communities, Department of Chemistry and Bioscience Aalborg University Aalborg Denmark; ^9^ Osaka International Research Center for Infectious Diseases Osaka Metropolitan University Sakai Japan

**Keywords:** comammox, methyl‐plastoquinones, nitrification, *Nitrospira*, respiration, reverse electron transport

## Abstract

Isoprenoid quinones are important compounds in most organisms. They are essential in electron and proton transport in respiratory and photosynthetic electron transport chains, and additional functions include oxidative stress defence. The biologically most relevant quinones are naphthoquinones including menaquinone and benzoquinones including ubiquinone and plastoquinone. They differ in their polar headgroup structures, physicochemical properties, and distribution among organisms. Menaquinone is the most widespread quinone in prokaryotes, ubiquinone occurs only in bacteria of the phylum *Pseudomonadota* and eukaryotes, and plastoquinone exists in phototrophic *Cyanobacteria* and plants. We found that chemolithoautotrophic nitrifying bacteria of the genus *Nitrospira* (phylum *Nitrospirota*) exclusively possess unusual methyl‐plastoquinones with a standard redox potential below that of canonical plastoquinone and ubiquinone but above menaquinone, suggesting functional roles in reverse electron transport, ammonia oxidation, alternative energy metabolisms, and oxidative stress mitigation. This extends the known diversity of quinones and suggests that plastoquinone derivatives are essential in ecologically important, non‐phototrophic bacteria.

## Graphical abstract

Chemolithoautotrophic nitrifying bacteria of the genus *Nitrospira* exclusively possess unusual methyl‐plastoquinones with a standard redox potential below that of canonical plastoquinone and ubiquinone, suggesting functional roles in bidirectional electron transport. These findings extend the known diversity of quinones and suggest that plastoquinone derivatives are essential in ecologically important, non‐phototrophic bacteria.

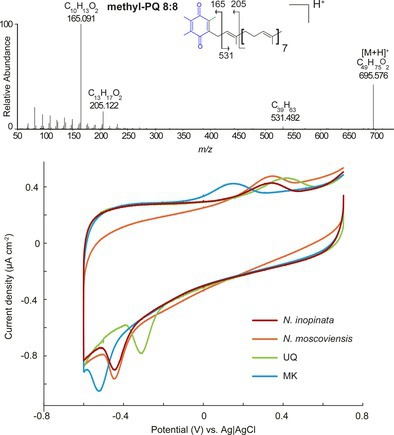

## 1 Introduction

Quinones have essential functions in energy metabolism across all domains of life (Nowicka and Kruk 2010). The major biologically relevant quinone classes are phylogenetically differentially distributed and have been used as chemotaxonomic molecular markers and biomarkers for profiling environmental microbial communities and microbially catalysed redox processes (Nowicka and Kruk 2010; Becker et al. 2018). Recent studies revealed an immense diversity of microorganisms (Hug et al. 2016) whose physiological and biochemical properties, such as their quinone types, are uncharacterised. Thus, still unidentified quinone forms may exist as adaptations to specific microbial metabolisms. More complete insights into quinone diversity and function will be pivotal to a deeper understanding of the biochemistry and evolution of electron transport chains in different physiological contexts.

Nitrification, the sequential oxidation of ammonia via nitrite to nitrate, is a major process of the biogeochemical nitrogen cycle. Aerobic, chemolithoautotrophic nitrite‐oxidising bacteria from the genus *Nitrospira* (phylum *Nitrospirota*) catalyse the second nitrification step in most ecosystems (Lücker et al. 2010). Recently, complete ammonia oxidizers (“comammox”), which oxidise both ammonia and nitrite, were identified in the genus *Nitrospira* (Daims et al. 2015; van Kessel et al. 2015). Like the canonical nitrite oxidizers, comammox *Nitrospira* are widespread across ecosystems. The energy metabolism of the canonical nitrite‐oxidising and comammox *Nitrospira* has been reconstructed from genomes (Lücker et al. 2010; Daims et al. 2015; van Kessel et al. 2015), although many details remain unresolved. One long‐standing open question had been the type of quinones in *Nitrospira*. These bacteria have homologues of respiratory complexes that obligately interact with quinones in other organisms, but no complete biosynthesis pathways of canonical ubi‐ or menaquinones had been identified in *Nitrospira* genomes. This unusual situation had not been addressed by chemical analyses of *Nitrospira* quinones. In two parallel independent studies, we and others (Elling et al. 2025) discovered and structurally characterised novel plastoquinone derivatives in *Nitrospira*. Elling et al. (2025) reconstructed a tentative biosynthesis pathway of these plastoquinone‐like compounds, analysed their distribution in the phylum *Nitrospirota*, and then focused on the evolution of respiratory quinones across the domains of life. We, on the other hand, determined the standard redox potential of the new plastoquinone‐like compounds and set these results in context with the unique, complete nitrification pathway in comammox and the nitrite‐oxidising metabolism in canonical *Nitrospira*.

## 2 Materials and Methods

### 2.1 Batch Cultivation, Cell Harvesting, and Purity Checks of *Nitrospira inopinata* and *Nitrospira moscoviensis* for Quinone Extraction

A pure culture of the comammox organism *Nitrospira inopinata* (Daims et al. 2015) was cultivated at 37°C in the dark and without agitation in liquid mineral medium containing (per litre): 0.6 g NaCl, 0.025 g MgCl_2_·6H_2_O, 0.01 g CaCl_2_·2H_2_O, 0.075 g KCl, 1 g HEPES [4‐(2‐hydroxyethyl)piperazine‐1‐ethanesulfonic acid]. Prior to autoclaving, the pH was adjusted to 8.2 with 1M NaOH. After autoclaving, 1 mL of sterile‐filtered trace element solution (TES, 1000×, per litre: 0.0344 g MnSO_4_·H_2_O, 0.05 g H_3_BO_3_, 0.07 g ZnCl_2_, 0.0726 g Na_2_MoO_4_·2H_2_O, 0.02 g CuCl_2_·2H_2_O, 0.024 g NiCl_2_·6H_2_O, 0.08 g CoCl_2_·6H_2_O, 1 g FeSO_4_·7H_2_O) and of selenium‐wolfram solution (SWS, 1000×, per litre: 0.003 g Na_2_SeO_3_·5H_2_O, 0.004 g Na_2_WO_4_, 0.0005 g NaOH), as well as KH_2_PO_4_ (50 mg/L), was added to the medium (Kits et al. 2017). The cultures were grown in 5 L Schott glass bottles with a minimal air headspace volume of 20% (in total six *N*. *inopinata* cultures were maintained). Ammonium was provided as the sole substrate by adding aliquots of a sterile, 1M NH_4_Cl stock solution to achieve a final concentration of 0.5–1.5 mM ammonium in the medium. The pH was determined regularly (~once per week), and NaHCO_3_ (from a 1M stock solution) was added as soon as the pH dropped below 7.8.

A pure culture of the canonical nitrite oxidizer *Nitrospira moscoviensis* (Ehrich et al. 1995) was cultivated as described elsewhere (Palatinszky et al. 2015). The cultures were grown in 5 L Schott glass bottles with a minimal air headspace volume of 20% (in total four *N. moscoviensis* cultures were maintained). Nitrite was provided as the sole substrate by adding aliquots of a sterile, 5M NaNO_2_ stock solution to achieve a final concentration of 5 mM nitrite in the medium.

During batch cultivation, *N. inopinata* and *N. moscoviensis* formed flocs that were visible by eye and settled at the bottom of the bottles. The flocs were harvested with a serological pipette and centrifuged in sterile 50 mL Falcon tubes at 16,639 × g for 30 min at 4°C. The biomass was then resuspended in sterile mineral medium (see above) and collected by centrifugation (20,817 × g, 30 min, 4°C) in 1.8 mL cryovials. The supernatant was discarded and the cell pellets were stored at −80°C. The frozen biomass was freeze‐dried (Alpha 1‐4 LSC Basic, Martin Christ Gefriertrocknungsanlagen GmbH) with default settings overnight and again stored at −80°C.

Immediately after floc harvesting, the remaining cultures were homogenised by shaking the bottles, and samples for contamination tests were collected. To check for contaminating heterotrophic microorganisms, 1 mL of each culture was inoculated into 5 mL of 0.1× Luria‐Bertani (LB) medium and incubated at 37°C without agitation. No heterotrophic growth was detected in any culture after 2 weeks of incubation. In addition, 3 mL aliquots from each *Nitrospira* culture were fixed in 3% formaldehyde as described elsewhere (Daims et al. 2005) and analysed by 16S ribosomal RNA‐targeted fluorescence in situ hybridisation (FISH) with the oligonucleotide probes Ntspa662 (specific for the genus *Nitrospira*) and the EUB338 probe mix (detects most bacteria) (Daims et al. 1999, 2001). The probes were labelled with different fluorochromes and applied simultaneously to the same samples. All cells in the samples were also counterstained with DAPI (4′,6‐diamidino‐2‐phenylindole). Upon inspection of the FISH probe‐specific and the DAPI fluorescence signals by epifluorescence microscopy, no microorganisms other than *Nitrospira* cells were observed. Furthermore, 1 mL aliquots from each culture bottle were centrifuged (20,817 × g, 20 min, room temperature). The cell pellets were stored at −20°C for further contamination checks by restriction fragment length polymorphism (RFLP) analysis. For RFLP, DNA was extracted using the innuPREP DNA Mini Kit (Analytik Jena) according to the manufacturer's instructions. 16S rRNA gene‐specific PCR was performed using the universal bacterial primers 616 V and 1492R [50°C annealing temperature, 35 cycles, DreamTaq polymerase (Thermo Scientific)]. The PCR products were purified using the innuPREP PCRpure Kit (Analytik Jena) and digested with the restriction enzymes AluI and Hin6I (Thermo Scientific) according to the manufacturer's protocol. The digestion products were separated by gel electrophoresis in a 2% agarose gel at 70 V for 90 min. The observed restriction fragment patterns were consistent with the fragments predicted from the 16S rRNA gene sequences of either *Nitrospira* species in all RFLP analyses, indicating the purity of the cultures.

After the first round of floc harvesting and purity checks, the remaining cultures (~17 L of *N. inopinata* and ~18 L of *N. moscoviensis* cultures) were further maintained for two to 3 weeks. Afterwards, the newly formed flocs were harvested as described above and the remaining liquid cultures were filtered through 0.22 μm pore‐size filters (Sartorius Sartolab 180E05‐E filtration unit). The biomass collected on the filters was scraped off, and the filters were washed twice in sterile medium to recover as many cells as possible. The biomass and wash solution were collected, centrifuged, and stored as described above. Aliquots for contamination tests were collected as described above. In total, 599 mg of *N. inopinata* and 356 mg of *N. moscoviensis* biomass (wet weight) were harvested.

### 2.2 Batch Cultivation, Cell Harvesting, and Purity Checks of *Nitrospira moscoviensis* for Voltammetric Quinone Analysis

Four additional pure cultures of *Nitrospira moscoviensis* were cultivated as described above but in 500 mL Schott glass bottles with a minimal air headspace volume of 20%. Nitrite was provided as the sole substrate by adding aliquots of a sterile, 1M NaNO_2_ stock solution to achieve a final concentration of 2 mM nitrite in the medium until a total of ~5 to 7 mM nitrite was consumed. Thereafter, the cultures were harvested by transferring 50 mL aliquots with a serological pipette into sterile 50 mL Falcon tubes and by centrifugation (16,639 × g, 30 min, 4°C). These steps were repeated until the entire culture volume (~350 mL) was pelleted. The supernatant was discarded after each centrifugation step, and the cell pellets were stored at −74°C. The frozen biomass was freeze‐dried (Alpha 1‐4 LSC Basic, Martin Christ Gefriertrocknungsanlagen, Germany) with default settings overnight and again stored at −74°C.

To check for contaminating heterotrophic microorganisms, 1 mL of each culture was weekly inoculated into 5 mL of 0.1× LB medium and incubated at 37°C without agitation. No heterotrophic growth was detected in any culture after 2 weeks of incubation.

### 2.3 Continuous Cultivation of *N. inopinata* for Voltammetric Quinone Analysis

A pure culture of *N. inopinata* was grown in a 23 L (18 L working volume) autoclavable glass vessel bioreactor system (Z310110012, Getinge Applikon, Delft, the Netherlands). Mineral medium (10 L) was prepared by diluting a concentrated (50×) stock solution with the following composition (L^−1^): 29.2 g NaCl, 3.75 g KCl, 0.55 g CaCl_2_·2H_2_O, 1.2 g MgSO_4_·7H_2_O. TES (1000×) and SWS (1000×) were added together with a 1M NH_4_Cl stock solution (final concentration, 2 mM) to the 1× mineral medium, which was sterilised in the 20 L reactor vessel by autoclaving. Finally, 100 mL of a 100× (5.44 g L^−1^) KH_2_PO_4_ solution was autoclaved separately and sterilely added to the medium. The pH sensor was calibrated before autoclaving, and the dissolved oxygen (dO_2_) sensor was calibrated after autoclaving per manufacturer instructions. Saturation of 100% dO_2_ was defined after sparging the 10 L of medium for a couple of hours with compressed air until a stable dO_2_ concentration was observed. Cultivation was initiated by sterile inoculation of 1 L of an active *N. inopinata* batch culture into the reactor vessel. During initial cultivation start‐up, the reactor was set to 37°C, agitation (stirring) remained off, medium was not supplemented, and no biomass was harvested until after the initial 2 mmol L^−1^ NH_4_^+^ was consumed. However, the pH and dO_2_ level were regulated manually to 7.8% and 20%, respectively. As soon as NH_4_^+^ was no longer detectable in the medium, reactor agitation was set to 50 rotations per minute, and continuous cultivation was initiated. Mineral medium (1×, complemented with 200 mL 100× KH_2_PO_4_, 20 mL 1000× TES, and 20 mL 1000× SeW) containing either 10 mM NH_4_Cl (first two harvests) or 20 mM NH_4_Cl (third and fourth harvests) was supplemented at a rate of 2 L per day, and the culture volume was maintained at 18 L. The pH was automatically kept at 7.8 via titration with sterile NaHCO_3_ (10 g L^−1^), resulting in a passive drip of 0.5 to 1 L per day, resulting in a final dilution rate of 0.14 to 0.17 (2.5 to 3 L per day). To check for contaminating heterotrophic microorganisms, 5 mL of liquid organic media (1:10 (v/v) diluted lysogeny broth and tryptic soy broth) were inoculated with 1 mL of reactor effluent. Additionally, effluent samples were formaldehyde fixed and inspected by FISH as described above for batch cultivation. No contaminations were detected at any time.

During continuous cultivation, reactor effluent was continuously collected but was harvested and processed in ~20 L batches every 7–8 days. A total of four harvests were conducted (~80 L of reactor effluent total). For each harvest, the biomass was concentrated to 200 mL using a tangential flow filtration system (Sartoflow Advanced, Sartorius Stedim Systems GmbH, Guxhagen, Germany). The cells were then further concentrated by three rounds of centrifugation (16,639 × g, 10 min, 22°C), producing four wet *N. inopinata* biomass samples of 300.4, 518.5, 552.5, and 895.6 mg. These were preserved at −74°C until further use.

### 2.4 Quinone Extraction and Structural Analysis

Quinones were extracted from freeze‐dried biomass using a modified Bligh–Dyer procedure (Bale et al. 2021). Briefly, the biomass was treated ultrasonically three times for 10 min with a solvent mixture of methanol, dichloromethane and phosphate buffer (2:1:0.8, v:v:v). After sonication, the combined supernatants were phase‐separated by adding additional dichloromethane and phosphate buffer to a final solvent ratio of 1:1:0.9 (v:v:v). The organic phase containing the intact polar lipids (IPLs) was collected and the aqueous phase re‐extracted two times with dichloromethane. The residue was then re‐extracted following the same procedure but starting with a solvent mix of methanol, dichloromethane and trichloroacetic acid pH 2–3 (2:1:0.8, v:v:v). Finally, the combined extract was dried under a stream of N_2_ gas. Before analysis, the extract was redissolved in a mixture of methanol: dichloromethane (9:1, v:v). Subsequently, aliquots were filtered through 0.45 μm regenerated cellulose syringe filters (4 mm diameter; Grace Alltech, Deerfield, IL, United States). Analysis was carried out using Ultra High Pressure Liquid Chromatography‐High Resolution Mass Spectrometry (UHPLC‐HRMS) according to the reversed phase method of Wörmer et al. (2013) with modifications as per Bale et al. (2021). We used an Agilent 1290 Infinity I UHPLC equipped with a temperature‐controlled auto‐injector and column oven, coupled to a Q Exactive Orbitrap MS with Ion Max source with heated electrospray ionisation (ESI) probe (Thermo Fisher Scientific, Waltham, MA, United States). Separation was achieved on an Acquity BEH C18 column (2.1 × 150 mm, 1.7 μm; Waters Corporation, Milford, MA, United States) maintained at 30°C. The eluent composition was (A) methanol/H_2_O/formic acid/14.8M NH_3aq_ (85:15:0.12:0.04 [v:v]) and (B) isopropanol/methanol/formic acid/14.8M NH_3aq_ (50:50:0.12:0.04 [v:v]). The elution program was: 5% B for 3 min, followed by a linear gradient to 60% B at 12 min and then to 100% B at 50 min; this was maintained until 80 min. The flow rate was 0.2 mL min^−1^. Positive ion electrospray ionisation (ESI) settings were: capillary temperature, 300°C; sheath gas (N_2_) pressure, 40 arbitrary units (AU); auxiliary gas (N_2_) pressure, 10 AU; spray voltage, 4.5 kV; probe heater temperature, 50°C; S‐lens 70 V. Target lipids were analysed with a mass range of *m/z* 350–2000 (resolving power 70,000 ppm at *m/z* 200), followed by data‐dependent mass spectrometry (MS^2^) (resolving power 17,500 ppm), in which the 10 most abundant masses in the mass spectrum were fragmented successively (stepped normalised collision energy 15, 22.5, 30; isolation width, 1.0 *m/z*). The MS was calibrated within a mass accuracy range of 1 ppm using the Thermo Scientific Pierce LTQ Velos ESI Positive Ion Calibration Solution. During analysis, dynamic exclusion was used to temporarily exclude masses (for 6 s) in order to allow selection of less abundant ions for MS^2^.

Compounds were quantified in terms of their MS peak area response. As different ions may exhibit different response behaviour, the relative abundance of the peak area does not necessarily reflect the actual relative abundance of the different compounds. The peak areas were determined from extracted ion chromatograms of the combined [M + H]^+^, [M + NH_4_]^+^ and [M + Na]^+^ ion (where present) for each individual quinone species. No known quinones were detected in the two *Nitrospira* species, based on accurate mass and characteristic fragment ions in MS^2^ (Elling et al. 2016). However, 10 components were detected that all gave distinctive quinone‐like MS^2^ spectra. These were compared with the MS^2^ spectra of plastoquinone 9:9 from *Chlorogloeopsis fritschii* str. PCC 6912, grown, extracted for lipids, and analysed as described in Gallego et al. (2024).

An aliquot of the Bligh Dyer extract was hydrogenated to aid in the identification of the unusual quinones. Hydrogenation was done in ethyl acetate (EtOAc) with one drop of concentrated acetic acid by bubbling H_2_ over PtO_2_ as catalyst (Aldrich) for 1 h at room temperature, followed by overnight stirring.

## 3 Voltammetric Quinone Characterisation and Standard Redox Potential Approximation

The approach used for the voltammetric analysis of quinones from desiccated microbial cells was established in a previous study (Le et al. 2015). The surface of an indium tin oxide (ITO) glass strip with dimensions of 26 × 77 mm^2^ (Geomatec Co. Ltd.) was covered with a UV‐cured insulating resin film using a Roland DG LEF12 inkjet printer, except for a circular area (diameter 4.0 mm) used as the working electrode (geometric area: 0.126 cm^2^). An Ag|AgCl|saturated KCl electrode and a platinum mesh electrode (1 × 1 cm) served as the reference and counter electrodes, respectively, throughout this study. The freeze‐dried biomass was dispersed in ultrapure water to prepare a suspension, and 10 μL of the suspension (~1 × 10^10^ cells mL^−1^) was applied to the exposed surface of the ITO electrode, which was then placed in a desiccator maintained at 298 K and 24% relative humidity until the surface was completely dry. Voltammograms were recorded in a pH 7.0 phosphate buffer at 20 mV s^−1^ of sweep rate under a nitrogen atmosphere in a Faraday cage thermostated at 303 K with a CHi842B Electrochemical Analyser. Obtained voltammograms were analysed using Wolfram Mathematica 10 software. To evaluate the voltammograms consisting of a sharp cathodic peak and a broad anodic peak resulting from interactions between the adsorbed species, and to infer the standard redox potentials of the quinones, simulations based on the Frumkin isotherm (Bard and Faulkner 2000) were performed on the bacterial samples following the procedure of Laviron (Laviron 1979; Laviron and Roullier 1980). This approach was described in detail in a previous study (Le et al. 2015). The obtained standard redox potentials versus the Ag|AgCl electrode were converted to potentials versus the standard hydrogen electrode (SHE) by adding 199 mV (the potential of the Ag|AgCl electrode vs. SHE is +199 mV at 298 K).

## 4 Results

To identify the quinones of *Nitrospira*, the only available comammox isolate *N. inopinata* and the nitrite‐oxidising isolate *N. moscoviensis* were grown for quinone analysis. It is noteworthy that the amounts of biomass needed to chemically characterise components of the bioenergetic metabolism are extremely difficult to obtain from slow‐growing *Nitrospira* isolates. To overcome this obstacle and obtain sufficient biomass for this study, we significantly upscaled our routine batch cultivation protocols for these bacteria, which usually use culture volumes between 250 and 750 mL, and grew the isolates of both species in multiple parallel batch cultures in 5 L vessels (total liquid volume, 16–24 L) with a harvesting strategy optimised for collecting floccular and suspended biomass with minimal loss. Additionally, we established continuous chemostat cultivation of *N. inopinata* (18 L working volume) that yielded up to 1 g (wet weight) per week of pure biomass grown under highly controlled conditions. The chemostat was operated for several months without a decrease in growth or detectable contamination of the culture, both of which are issues that frequently hamper the long‐term, uninterrupted cultivation of nitrifier isolates.

**FIGURE 1:**
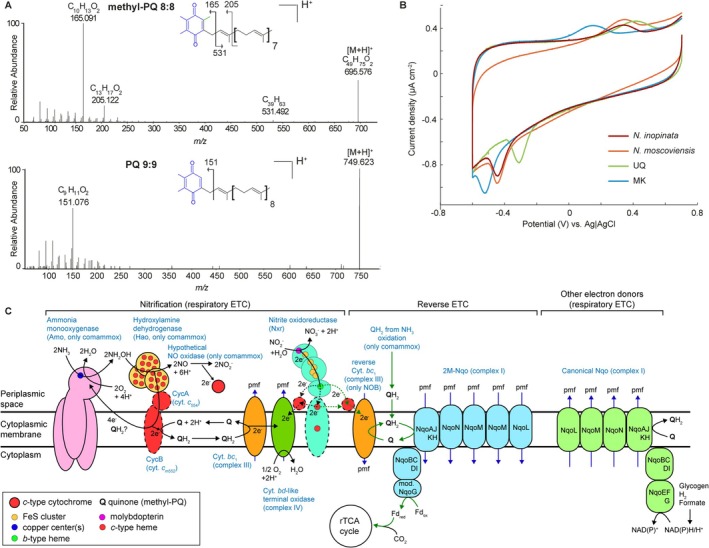
Characterisation and potential physiological roles of methyl‐PQs in *Nitrospira*. (A) Mass spectrometry spectra of methyl‐PQ 8:8 from *N. inopinata* and of canonical plastoquinone 9:9 from *Chlorogloeopsis fritschii* . The headgroups are highlighted in blue and the additional methyl in green. (B) Voltammograms of *N. inopinata* and *N. moscoviensis* cells, representing the methyl‐PQs, and of pure ubiquinone (UQ) and menaquinone (MK) for comparison. (C) Genome‐based model of the membrane‐bound respiratory and reverse electron transport chains in comammox and nitrite‐oxidising *Nitrospira*. Key nitrification enzymes and respiratory complexes are shown. Black arrows indicate electron transport in respiratory electron transport chains, green arrows the reverse electron transport chain, and blue arrows proton translocation across the cytoplasmic membrane. Dashed contours indicate uncertain function of a protein, and dashed lines indicate uncertain electron transfers. At present, the details of electron flow from nitrite oxidoreductase to complexes III and IV via soluble or membrane‐bound *c*‐type cytochromes remain uncertain (Lücker et al. 2010; Mundinger et al. 2019). Complex IV of *Nitrospira* belongs to the cytochrome *bd*‐type oxygen reductase superfamily but lacks a quinol binding site and likely uses *c*‐type cytochromes as alternative electron donors (Lücker et al. 2010; Murali et al. 2021). ETC, electron transport chain; Cyt., cytochrome; Fd, ferredoxin; NOB, nitrite‐oxidising bacteria; Q quinone; other abbreviations see text.

Neither canonical ubiquinone nor menaquinone was found in the extracts, but nine components detected in *N. inopinata* gave distinctive quinone‐like mass spectrometry spectra (Table 1). Further analysis identified these components as novel methyl‐plastoquinones (methyl‐PQs) with different lengths and degrees of unsaturation of the isoprenoidal chains (Table 1, Figure 1A, Supplementary Results).

**TABLE 1:** Quinone composition of *N. inopinata* and *N. moscoviensis* .

Quinone	Accurate mass (*m/z*)	AEC	Δ mmu	Relative abundance (%)	
				*N. inopinata*	*N. moscoviensis*
Methyl‐PQ 6:6	559.451	C_39_H_59_O_2_	1.1	1.6	n.d.
Methyl‐PQ 6:5	561.467	C_39_H_61_O_2_	1.5	0.3	n.d.
Methyl‐PQ 7:7	627.515	C_44_H_67_O_2_	0.9	**40**	0.1
Methyl‐PQ 7:6	629.530	C_44_H_69_O_2_	0.9	1.4	n.d.
Methyl‐PQ 8:8	695.577	C_49_H_75_O_2_	0.5	**45.3**	2.1
Methyl‐PQ 8:7	697.592	C_49_H_77_O_2_	0.1	**10.8**	n.d.
Methyl‐PQ 9:9	763.639	C_54_H_83_O_2_	0.2	0.6	**67.1**
Methyl‐PQ 9:8	765.654	C_54_H_85_O_2_	0.2	0.1	n.d.
Methyl‐PQ 10:10	831.701	C_59_H_91_O_2_	0.0	< 0.1	**29.2**
Methyl‐PQ 11:11	899.764	C_64_H_99_O_2_	0.3	n.d.	1.5

*Note:* Methyl‐PQs with a relative abundance above 10% in one of the species are highlighted in boldface.

Abbreviations: AEC, assigned elemental composition; mmu, milli mass unit; Δ mmu = (measured mass − calculated mass) × 1000. All AECs represent an [M + H]^+^ ion; n.d., not detected.

Remarkably, the unusual methyl‐PQs were the only quinones detected in both *Nitrospira* species. These results are consistent with recently published findings of Elling et al. (2025) and provide independent confirmation for the unexpected presence of these novel plastoquinones containing an additional methyl group in *Nitrospira*. Still, their nomenclature needs clarification: According to IUPAC rules, in the fully substituted benzoquinone ring the longest (i.e., the isoprenoid chain) substituent is given locant 2, the novel methyl substituent locant 3, and the two methyl substituents also present in canonical plastoquinone are assigned locants 5 and 6. Thus, IUPAC nomenclature (Supplementary Results) uses different locant assignments than Elling et al. (2025).

The redox potential is a key physicochemical parameter and determines how quinones can be involved in electron transport in an organism. Especially, it determines with which electron donors and acceptors the quinones may interact in the electron transport chains. To elucidate possible physiological functions of the methyl‐PQs in *Nitrospira*, we applied voltammetry for the redox profiling of quinones from desiccated microbial cells (Le et al. 2015). This approach uses relatively small amounts of biomass and is well‐suited for characterising the quinones of slow‐growing microorganisms that do not reach high cell densities in culture. Interestingly, the peak potentials in the voltammograms of *Nitrospira* differed substantially from those of bacteria containing menaquinone or ubiquinone (Le et al. 2015) and from purified mena‐ and ubiquinone (Figure 1B, Dataset S1). The standard redox potential (*E*°′) of the methyl‐PQs was found to be 57 mV (vs. standard hydrogen electrode) for *N. inopinata* and 59 mV for *N. moscoviensis* . This lower *E*°′ compared to ubiquinone and canonical plastoquinone (both, *E*°′≈100 mV) (Schoepp‐Cothenet et al. 2013) is fully consistent with a reduction of *E*°′ by 40–80 mV for each additional methyl substituent at the headgroup in other quinones (Frontana et al. 2006; Wilkens and Simon 2023).

## 5 Discussion

Quinones function as electron donors and acceptors in electron transport chains (Schoepp‐Cothenet et al. 2013). Canonical plastoquinones are essential for oxygenic photosynthesis (Nowicka and Kruk 2010), but the non‐phototrophic *Nitrospira* conserve energy by aerobic respiration. In respiratory electron transport chains, canonical plastoquinone (only in *Cyanobacteria*), ubiquinone (both, *E*°′≈100 mV), and menaquinone (*E*°′≈−70 mV) (Schoepp‐Cothenet et al. 2013) accept electrons from donors with redox potentials below that of the quinones in electron transfer steps which are thermodynamically favourable. However, *Nitrospira* utilise high‐potential electron donors, ammonia and nitrite [*E*°′ (NO_2_^−^/NH_3_) = 330 mV; *E*°′ (NO_3_^−^/NO_2_^−^) = 430 mV]. Consequently, quinones are not involved in the respiratory electron transport chain for nitrite oxidation (Lücker et al. 2010) (Figure 1C). Only in ammonia‐oxidising comammox *Nitrospira*, electrons from hydroxylamine could be fed into the quinone pool and may be used for respiration or be channelled back to the electron‐requiring ammonia monooxygenase (Figure 1C), as proposed for ammonia‐oxidising bacteria that use ubiquinone (Simon and Klotz 2013). Since methyl‐PQs are the only quinones in comammox and nitrite‐oxidising *Nitrospira*, we hypothesise that they must have important functions beyond respiration. *Nitrospira* fix CO_2_ via the reductive tricarboxylic acid (rTCA) cycle (Lücker et al. 2010). They depend on reverse electron transport to reduce low‐potential (*E*°′ ≤ −500 mV) ferredoxins, which are key redox partners in the rTCA cycle (Li and Elliott 2016), with electrons from ammonia or nitrite. Reverse electron transport involves thermodynamically unfavourable electron transfer steps driven by energy consumption, such as consumption of proton motive force, and quinones can act as electron carriers also in the reverse direction (Wilkens and Simon 2023). At the onset of reverse electron transport in nitrite‐oxidising *Nitrospira*, electrons stemming from nitrite are transferred to quinone (Q) by a proton‐pumping Q‐cytochrome *c* oxidoreductase (complex III) that works in reverse, consuming proton motive force (Lücker et al. 2010) (Figure 1C). This reaction should be facilitated by a high redox potential of the electron‐accepting quinone. The next reverse electron transport step in all *Nitrospira* likely involves a NADH‐quinone oxidoreductase (2M‐Nqo; complex I) with two instead of only one NqoM proton‐pumping subunits. This unusual 2M‐Nqo lacks a NAD(H) binding site and may transfer electrons from quinone to ferredoxin at the expense of proton motive force (Chadwick et al. 2018) (Figure 1C), which should be facilitated by a low redox potential of the electron‐donating quinone. The methyl‐PQs, owing to their intermediate redox potential (*E*°′≈50–60 mV) between ubiquinone and menaquinone, may be suitable redox partners in both reverse electron transport steps (Figure 1C).

Many *Nitrospira* members possess alternative energy metabolisms using H_2_ or formate (Koch et al. 2014, 2015) and form glycogen as a storage compound (Lücker et al. 2010; Daims et al. 2015; Koch et al. 2015). In the absence of other quinones, the methyl‐PQs are likely also part of the respiratory electron transport chain with such low‐potential electron donors, which involves a canonical Nqo and complex III also found in all *Nitrospira* (Figure 1C).

Due to a lack of sufficient biomass from *Nitrospira* or any other aerobic *Nitrospirota* member, Elling et al. (2025) did not measure the redox potential of the methyl‐PQs but instead calculated it using density functional theory. These calculations resulted in an estimated redox potential below that of canonical plastoquinone and above that of ubiquinone (Elling et al. 2025). Such a high redox potential would most likely not allow for the essential role of these quinones as electron carriers between enzyme complexes of the reverse electron transport chain, as discussed above. In contrast, our voltammetric measurements revealed that the methyl‐PQs have a lower standard redox potential than canonical plastoquinone and ubiquinone (but still higher than menaquinone), which likely allows them to function in reverse electron transport as well as in aerobic respiratory electron transport. During aerobic respiration, they accept electrons from hydroxylamine in comammox or from low‐potential electron donors in *Nitrospira* cells using alternative energy metabolisms or degrading storage compounds (Figure 1C). Thus, the methyl‐PQs represent a molecular adaptation to the bioenergetic challenges of bidirectional electron transport in chemolithoautotrophs that use high‐potential electron donors to (i) conserve energy and (ii) fuel their intracellular reductant pool for CO_2_ fixation.

In addition to their functional roles in electron transport, the methyl‐PQs may facilitate the survival of *Nitrospira* in oxic conditions. As is the case for other benzoquinones, their reduced forms (methyl‐PQH_2_) should be less sensitive than reduced menaquinone to non‐enzymatic oxidation by O_2_ (Nowicka and Kruk 2010). Canonical plastoquinones are efficient scavengers of reactive oxygen species (Nowicka and Kruk 2010). The methyl‐PQs may share this property, which would be beneficial to *Nitrospira* species that often lack catalase and/or superoxide dismutase (Lücker et al. 2010; Daims et al. 2015).

Experimental verification of these hypotheses on the physiological roles of the novel methyl‐PQs in *Nitrospira* will be challenging. Enough biomass for more detailed biochemical studies is difficult and very expensive to obtain from the extremely slow‐growing *Nitrospira* cultures; the heterologous expression of *Nitrospira* membrane enzyme complexes has not been successful yet, and *Nitrospira* are not yet amenable to genetic manipulation. Therefore, we can currently also not assign specific functions to the different methyl‐PQ compounds detected in the two *Nitrospira* species (Table 1). However, our findings provide a basis for dedicated follow‐up research on the unusual quinones of *Nitrospira*, which might also become useful biomarkers for these bacteria in the environment. Further upscaling the *Nitrospira* cultures to volumes above 100 L could provide the biomass required for in‐depth biochemistry and structural biology research and, although challenging, does not appear to be entirely unrealistic based on the improved cultivation protocols developed in this study.

We conclude that the range of known quinone structures, and the known distribution of plastoquinone‐like compounds in living organisms, are extended by methyl‐PQs that unexpectedly occur in non‐phototrophic bacteria and likely are essential for the bidirectional electron transport in nitrifiers of the genus *Nitrospira* in virtually all ecosystems.

## Author Contributions

N.J.B., M.K., J.S.S.D. performed quinone structure analyses; H.F., H.I., S.I., Y.Y. performed voltammetric analyses; J.P., C.J.S., H.P., P.P. cultured and prepared *Nitrospira* biomass; H.D., N.J.B., H.S., J.S.S.D., P.P., M.W. designed and supervised research; H.D. and N.J.B. wrote the paper. All authors contributed to reviewing and editing the manuscript.

## Conflicts of Interest

The authors declare no conflicts of interest.

## Supporting information


**Data S1:** Supporting Information.

**Data S2:** Supporting Information.

## Data Availability

The raw (.raw) mass spectrometry data are available through the Zenodo repository (DOI: 10.5281/zenodo.15590553). Data S1 includes the cyclic voltammetry raw data.

## References

[emi470174-bib-0001] Bale, N. J. , S. Ding , E. C. Hopmans , et al. 2021. “Lipidomics of Environmental Microbial Communities. I: Visualization of Component Distributions Using Untargeted Analysis of High‐Resolution Mass Spectrometry Data.” Frontiers in Microbiology 12: 659302. 34367080 10.3389/fmicb.2021.659302 PMC8343106

[emi470174-bib-0002] Bard, A. J. , and L. R. Faulkner . 2000. Electrochemical Methods: Fundamentals and Applications. Wiley.

[emi470174-bib-0003] Becker, K. W. , F. J. Elling , J. M. Schröder , et al. 2018. “Isoprenoid Quinones Resolve the Stratification of Redox Processes in a Biogeochemical Continuum From the Photic Zone to Deep Anoxic Sediments of the Black Sea.” Applied and Environmental Microbiology 84: e02736‐17. 29523543 10.1128/AEM.02736-17 PMC5930376

[emi470174-bib-0004] Chadwick, G. L. , J. Hemp , W. W. Fischer , and V. J. Orphan . 2018. “Convergent Evolution of Unusual Complex I Homologs With Increased Proton Pumping Capacity: Energetic and Ecological Implications.” ISME Journal 12: 2668–2680. 29991762 10.1038/s41396-018-0210-1 PMC6194058

[emi470174-bib-0005] Daims, H. , A. Brühl , R. Amann , K.‐H. Schleifer , and M. Wagner . 1999. “The Domain‐Specific Probe EUB338 Is Insufficient for the Detection of all *Bacteria*: Development and Evaluation of a More Comprehensive Probe Set.” Systematic and Applied Microbiology 22: 434–444. 10553296 10.1016/S0723-2020(99)80053-8

[emi470174-bib-0006] Daims, H. , E. V. Lebedeva , P. Pjevac , et al. 2015. “Complete Nitrification by *Nitrospira* Bacteria.” Nature 528: 504–509. 26610024 10.1038/nature16461 PMC5152751

[emi470174-bib-0007] Daims, H. , J. L. Nielsen , P. H. Nielsen , K. H. Schleifer , and M. Wagner . 2001. “In Situ Characterization of *Nitrospira*‐Like Nitrite‐Oxidizing Bacteria Active in Wastewater Treatment Plants.” Applied and Environmental Microbiology 67: 5273–5284. 11679356 10.1128/AEM.67.11.5273-5284.2001 PMC93301

[emi470174-bib-0008] Daims, H. , K. Stoecker , and M. Wagner . 2005. “Fluorescence *in Situ* Hybridisation for the Detection of Prokaryotes.” In Molecular Microbial Ecology, edited by A. M. Osborn and C. J. Smith , 213–239. Bios‐Garland.

[emi470174-bib-0009] Ehrich, S. , D. Behrens , E. Lebedeva , W. Ludwig , and E. Bock . 1995. “A New Obligately Chemolithoautotrophic, Nitrite‐Oxidizing Bacterium, *Nitrospira moscoviensis* sp. Nov. and Its Phylogenetic Relationship.” Archives of Microbiology 164: 16–23. 7646315 10.1007/BF02568729

[emi470174-bib-0010] Elling, F. J. , K. W. Becker , M. Könneke , et al. 2016. “Respiratory Quinones in *Archaea*: Phylogenetic Distribution and Application as Biomarkers in the Marine Environment.” Environmental Microbiology 18: 692–707. 26472620 10.1111/1462-2920.13086

[emi470174-bib-0011] Elling, F. J. , F. Pierrel , S.‐C. Chobert , et al. 2025. “A Novel Quinone Biosynthetic Pathway Illuminates the Evolution of Aerobic Metabolism.” Proceedings of the National Academy of Sciences 122: e2421994122. 10.1073/pnas.2421994122 PMC11874023 39977315

[emi470174-bib-0012] Frontana, C. , Á. Vázquez‐Mayagoitia , J. Garza , R. Vargas , and I. González . 2006. “Substituent Effect on a Family of Quinones in Aprotic Solvents: An Experimental and Theoretical Approach.” Journal of Physical Chemistry A 110: 9411–9419. 16869691 10.1021/jp060836+

[emi470174-bib-0013] Gallego, R. P. , F. A. B. von Meijenfeldt , N. J. Bale , J. S. S. Damsté , and L. Villanueva . 2024. “Emergence and Evolution of Heterocyte Glycolipid Biosynthesis Enabled Specialized Nitrogen Fixation in Cyanobacteria.” Preprint. bioRxiv. 10.1101/2024.05.17.594646. PMC11804610 39869795

[emi470174-bib-0014] Hug, L. A. , B. J. Baker , K. Anantharaman , et al. 2016. “A New View of the Tree of Life.” Nature Microbiology 1: 1–6. 10.1038/nmicrobiol.2016.48 27572647

[emi470174-bib-0015] Kits, K. D. , C. J. Sedlacek , E. V. Lebedeva , et al. 2017. “Kinetic Analysis of a Complete Nitrifier Reveals an Oligotrophic Lifestyle.” Nature 549: 269–272. 28847001 10.1038/nature23679 PMC5600814

[emi470174-bib-0016] Koch, H. , A. Galushko , M. Albertsen , et al. 2014. “Growth of Nitrite‐Oxidizing Bacteria by Aerobic Hydrogen Oxidation.” Science 345: 1052–1054. 25170152 10.1126/science.1256985

[emi470174-bib-0017] Koch, H. , S. Lücker , M. Albertsen , et al. 2015. “Expanded Metabolic Versatility of Ubiquitous Nitrite‐Oxidizing Bacteria From the Genus *Nitrospira* .” Proceedings of the National Academy of Sciences of the United States of America 112: 11371–11376. 26305944 10.1073/pnas.1506533112 PMC4568715

[emi470174-bib-0018] Laviron, E. 1979. “A.C. Polarography and Faradaic Impedance of Strongly Adsorbed Electroactive Species: Part II. Theoretical Study of a Quasi‐Reversible Reaction in the Case of a Frumkin Isotherm.” Journal of Electroanalytical Chemistry and Interfacial Electrochemistry 105: 25–34.

[emi470174-bib-0019] Laviron, E. , and L. Roullier . 1980. “General Expression of the Linear Potential Sweep Voltammogram for a Surface Redox Reaction With Interactions Between the Adsorbed Molecules: Applications to Modified Electrodes.” Journal of Electroanalytical Chemistry and Interfacial Electrochemistry 115: 65–74.

[emi470174-bib-0020] Le, D. Q. , A. Morishita , S. Tokonami , et al. 2015. “Voltammetric Detection and Profiling of Isoprenoid Quinones Hydrophobically Transferred From Bacterial Cells.” Analytical Chemistry 87: 8416–8423. 26218886 10.1021/acs.analchem.5b01772

[emi470174-bib-0021] Li, B. , and S. J. Elliott . 2016. “The Catalytic Bias of 2‐Oxoacid:Ferredoxin Oxidoreductase in CO_2_: Evolution and Reduction Through a Ferredoxin‐Mediated Electrocatalytic Assay.” Electrochimica Acta 199: 349–356.

[emi470174-bib-0022] Lücker, S. , M. Wagner , F. Maixner , et al. 2010. “A *Nitrospira* Metagenome Illuminates the Physiology and Evolution of Globally Important Nitrite‐Oxidizing Bacteria.” Proceedings of the National Academy of Sciences of the United States of America 107: 13479–13484. 20624973 10.1073/pnas.1003860107 PMC2922143

[emi470174-bib-0023] Mundinger, A. B. , C. E. Lawson , M. S. M. Jetten , H. Koch , and S. Lücker . 2019. “Cultivation and Transcriptional Analysis of a Canonical *Nitrospira* Under Stable Growth Conditions.” Frontiers in Microbiology 10: 1325. 31333593 10.3389/fmicb.2019.01325 PMC6606698

[emi470174-bib-0024] Murali, R. , R. B. Gennis , and J. Hemp . 2021. “Evolution of the Cytochrome *Bd* Oxygen Reductase Superfamily and the Function of CydAA’ in Archaea.” ISME Journal 15: 3534–3548. 34145390 10.1038/s41396-021-01019-4 PMC8630170

[emi470174-bib-0025] Nowicka, B. , and J. Kruk . 2010. “Occurrence, Biosynthesis and Function of Isoprenoid Quinones.” Biochimica et Biophysica Acta (BBA) 1797: 1587–1605. 20599680 10.1016/j.bbabio.2010.06.007

[emi470174-bib-0026] Palatinszky, M. , C. Herbold , N. Jehmlich , et al. 2015. “Cyanate as an Energy Source for Nitrifiers.” Nature 524: 105–108. 26222031 10.1038/nature14856 PMC4539577

[emi470174-bib-0027] Schoepp‐Cothenet, B. , R. van Lis , A. Atteia , et al. 2013. “On the Universal Core of Bioenergetics.” Biochimica et Biophysica Acta (BBA) 1827: 79–93. 22982447 10.1016/j.bbabio.2012.09.005

[emi470174-bib-0028] Simon, J. , and M. G. Klotz . 2013. “Diversity and Evolution of Bioenergetic Systems Involved in Microbial Nitrogen Compound Transformations.” Biochimica et Biophysica Acta (BBA) 1827: 114–135. 22842521 10.1016/j.bbabio.2012.07.005

[emi470174-bib-0029] van Kessel, M. A. H. J. , D. R. Speth , M. Albertsen , et al. 2015. “Complete Nitrification by a Single Microorganism.” Nature 528: 555–559. 26610025 10.1038/nature16459 PMC4878690

[emi470174-bib-0030] Wilkens, D. , and J. Simon . 2023. “Chapter One ‐ Biosynthesis and Function of Microbial Methylmenaquinones.” In Advances in Microbial Physiology, edited by R. K. Poole and D. J. Kelly , 1–58. Academic Press. 10.1016/bs.ampbs.2023.05.002 37507157

[emi470174-bib-0031] Wörmer, L. , J. S. Lipp , J. M. Schröder , and K.‐U. Hinrichs . 2013. “Application of Two New LC–ESI–MS Methods for Improved Detection of Intact Polar Lipids (IPLs) in Environmental Samples.” Organic Geochemistry 59: 10–21.

