# Supplementary material for: Unusual Plastoquinones in Non‐Phototrophic Nitrifying Bacteria
Source: Environ Microbiol Rep. 2025 Aug 5;17(4):e70174. doi: 10.1111/1758-2229.70174 (PMC12324825; doi:10.1111/1758-2229.70174)
Supplement: Supplementary file 2 — Data S2: Supporting Information. [file EMI4-17-e70174-s002.pdf]

Supporting Information for

Unusual Plastoquinones in Non-Phototrophic Nitrifying Bacteria

Nicole J. Bale, Hayato Fujimura, Petra Pjevac, Michel Koenen, Hikaru Ikeda, Satohiro Itagaki, Yojiro Yamamoto, Johanna Palmetzhofer, Christopher J. Sedlacek, Hayk Palabikyan, Jaap S. Sinninghe Damsté, Michael Wagner, Hiroshi Shiigi, Holger Daims

This PDF includes:

Supplemental Results

Legend for Dataset S1

Supplemental References

Other supporting materials for this manuscript include the following:

Dataset S1

Supplemental Results

Structural characterization of novel plastoquinones from *Nitrospira*.

Upon ultra high pressure liquid chromatography-high resolution mass spectrometry (UHPLC-HRMS) analysis all unknown, quinone-like components identified in the extracts from *N. inopinata* exhibited characteristic fragment ions at *m/z* 205.122 (minor) and *m/z* 165.091 (major) (main text Fig. 1A). Beyond these dominant fragments, the overall pattern of ions in the mass spectrometry (MS^2) spectra were similar to those of ubiquinones. For example, the MS^2 spectrum of the major unknown quinone-like-component with an $[M+H]^+$ at *m/z* 695.576 (main text Fig. 1A) contained the same distribution of isoprenoid fragments as that of canonical ubiquinone 8:8 analyzed in parallel for comparison (data not shown), such as: *m/z* 81.070 (C_6H_9), *m/z* 95.086 (C_7H_{11}), *m/z* 109.102 (C_8H_{13}), *m/z* 123.117 (C_9H_{15}), *m/z* 137.13251 ($C_{10}H_{17}$). Both also contained an ion at *m/z* 531.492 ($C_{39}H_{63}$), associated with a fully unsaturated, 8 isoprenoid unit chain. As this indicated that ubiquinone 8:8 and the unknown quinone-like component both contained the same unsaturated, 8 isoprenoid unit chain, this presumably left the unknown component with a $C_{10}H_{13}O_2$ (*m/z* 165.091) conjugated cyclic component, in place of the $C_{10}H_{13}O_4$ (*m/z* 197.081) present in ubiquinone. Hence, the unknown quinone-like component could be a plastoquinone with an additional CH_2. To test this structural assignment, the extracts were hydrogenated and again analyzed by MS. Hydrogenation of ubiquinone 8:8 led to the saturation of all eight double bonds of the isoprenoidal chain, resulting in a component with an $[M+H]^+$ ion at *m/z* 743.691 (*m/z* 727.566 + 16H), but left the 2,3-dimethoxy-5-methylbenzoquinone nucleus intact (the same fragment ion at *m/z* 197.081, [$C_{10}H_{13}O_4$]). Hydrogenation of the supposed plastoquinone homologue yielded comparable results. Its hydrogenated counterpart gave an $[M+H]^+$ ion at *m/z* 711.702 (*m/z* 695.577 + 16H) and, as per the hydrogenated ubiquinone 8:8, the cyclohexa-2,5-diene-1,4-dione nucleus containing three additional C atoms remained intact (fragment ion at *m/z* 165.091, [$C_{10}H_{13}O_2$]). The spectra of the putative plastoquinone-like components from *Nitrospira* were also compared with the MS^2 spectrum of canonical plastoquinone 9:9 from the cyanobacterium *Chlorogloeopsis fritschii* str. PCC 6912 that was grown, extracted for lipids, and analyzed as described elsewhere (Gallego *et al.*, 2024). The fragment at *m/z* 165.091 resembled that present in the spectrum of the cyanobacterial plastoquinone (*m/z* 151.075, $C_9H_{11}O_2$, main text Fig. 1A) except being 14 Da larger. This supported our notion that the quinone-like component is a plastoquinone with an additional CH_2 moiety. The absence of M-15 (loss of a methyl group loss from the parent ion) indicated that the additional CH_2 moiety is in the form of an additional methyl group on the remaining free position of the plastoquinone moiety, as an ethyl substituent

would be noticed during MS^2 fragmentation by a distinct M-15 fragment ion. Hence, the additional methyl must be at the unsubstituted ring position of the quinone moiety, analogous to the position of the only methyl group of ubiquinones (which has two methoxy substituents). Hence, the methyl PQ was identified as 3,5,6-trimethyl-2-[(2E,6E,10E,14E,18E,22E,26E)-3,7,11,15,19,23,27,31-octamethyldotriaconta-2,6,10,14,18,22,26,30,34-octaen-1-yl]cyclohexa-2,5-diene-1,4-dione. This identification is fully in line with that recently reported by Elling *et al.*, 2025.

Dataset S1

Dataset with the raw data of voltammetric analysis, which are shown in Figure 1B in the main text.

References

Elling, F.J., Pierrel, F., Chobert, S.-C., Abby, S.S., Evans, T.W., Reveillard, A., et al. (2025) A novel quinone biosynthetic pathway illuminates the evolution of aerobic metabolism. *Proc Natl Acad Sci* **122**: e2421994122.

Gallego, R.P., von Meijenfeldt, F.A.B., Bale, N.J., Damsté, J.S.S., and Villanueva, L. (2024) Emergence and evolution of heterocyte glycolipid biosynthesis enabled specialized nitrogen fixation in cyanobacteria. Preprint. bioRxiv: DOI 10.1101/2024.05.17.594646.
